# Blocking microtubule deacetylation inhibits anaphase chromosome movements in crane-fly spermatocytes

**DOI:** 10.1371/journal.pone.0311691

**Published:** 2024-12-02

**Authors:** Maral Janan, Jess MacPherson, Arthur Forer

**Affiliations:** Biology Department, York University, North York, Ontario, Canada; Centre de Recherche en Biologie cellulaire de Montpellier, FRANCE

## Abstract

Chromosome movement speeds during anaphase are regulated by depolymerization of microtubules. Several models describe chromosome movement during cell division but none of them consider post-translational modifications of tubulin, even though such modifications help specify microtubules for unique cellular activities. Among these modifications, acetylation of Lysine 40 is one of the common post-translational modifications. Acetylation of microtubules greatly improves their stability, especially when subjected to cooling or drug treatment. Since kinetochore microtubules are acetylated in a variety of eukaryote cells, we wondered whether deacetylation of kinetochore microtubules was necessary in order for microtubules to be able to depolymerize during anaphase. HDAC6 (Histone Deacetylase 6) deacetylates acetylated tubulin. To study whether tubulin must be deacetylated during anaphase, we added to living cells two different HDAC6 inhibitors (Tubacin and Trichostatin A), separately, as chromosomes moved poleward in anaphase. Both HDAC6 inhibitors altered chromosome movement: chromosomes either completely stopped moving, or moved more slowly, or sometimes continued movement without speed changes. The effects of the inhibitors on chromosome movement are reversible: half-bivalents either restarted anaphase movement by themselves before washing out the inhibitor or resumed their poleward movement after the inhibitor was washed out. We suggest that kinetochore microtubules need to be deacetylated in order for normal anaphase movements to occur.

## Introduction

Our experiments deal with acetylation of mitotic microtubules in cell division and its effect on microtubule depolymerization. It is generally agreed that when the chromosomes line up at the metaphase plate they are kept in the equatorial position by equal and opposite forces along the kinetochore microtubules that connect each chromosome to the opposite two poles, and that during metaphase the tubulin subunits enter the microtubules at the kinetochore and migrate (flux) along the microtubules to the pole. The various models of how chromosomes move during anaphase include force production generated by microtubule shortening at the kinetochore end or at the pole end [1,2] or by pushing by spindle matrix or actin or other forces external to the microtubules [3–5]. Regardless of which model is correct, all models recognise that the microtubules between chromosomes and poles depolymerize at one of the two ends, or both, and that depolymerization of microtubules is the rate-limiting step for chromosome movement. None of the movement models, however, consider post-translational modifications of tubulin. Tubulin post-translational modifications affect microtubule functions by directly modifying mechanical characteristics, or by influencing interactions with other proteins, or by modifying stability [6]. For example, Zadra et al. (2022) show that the enzyme TTLL11 (tubulin tyrosine ligase like 11) is crucial for microtubule polyglutamylation such that when TTLL11 is experimentally reduced microtubule stability is altered (they become “stronger”) which in turn causes altered chromosome segregation in mitosis [7].

Several classes of tubulin post-translational modifications are known, including tyrosination, acetylation, glutamylation, and glycylation [8]. Acetylation of α-tubulin (on α K40) is the most common post-translational modification of tubulin. Most tubulin post-translational modification sites are located on the outer surface of the microtubule, but tubulin acetylation occurs in the microtubule lumen [9]. Luminal tubulin acetylation induces a local structural rearrangement of αK40 and decreases lateral contact between microtubule protofilaments; reduction of lateral contact weakens inter-protofilament interactions, increases the flexibility of the microtubule against mechanical stress, and increases microtubule stability [10].

There is a time-lag between polymerisation of microtubules from tubulin subunits and when the microtubule subunits become acetylated; it takes minutes before newly polymerised microtubules become acetylated [11]. Most microtubules are not stable: they are dynamic, causing them to alternate between growth and shrinkage for different functions in a cell [12]. Acetylated microtubules are long-lived and usually are stable and ‘stronger’ than other microtubules in being resistant to drugs and to cold, both of which quickly depolymerise non-acetylated microtubules [13]. Microtubule acetylation is carried out by α-tubulin acetyltransferase (αTAT1). The acetylation is reversible with deacetylation occurring either via Histone Deacetylase 6 (HDAC6) or via NAD^+^-dependent deacetylase, silent information regulator 2 (SIRT2) [14]. HDAC6 is a microtubule-associated deacetylase; it is present in the cytoplasm and spindle and regulates the ensuing overall acetylation levels [15]. Overexpression of HDAC6 greatly decreases the level of tubulin acetylation [16]. SIRT2 is a nucleo‑cytoplasmic shuttling protein which is predominantly in the cytoplasm. Inhibiting SIRT2, as well as HDAC6, results in an increase in α-tubulin acetylation; however, the two deacetylation enzymes have different gene expression profiles [17].

**Fig 1:**
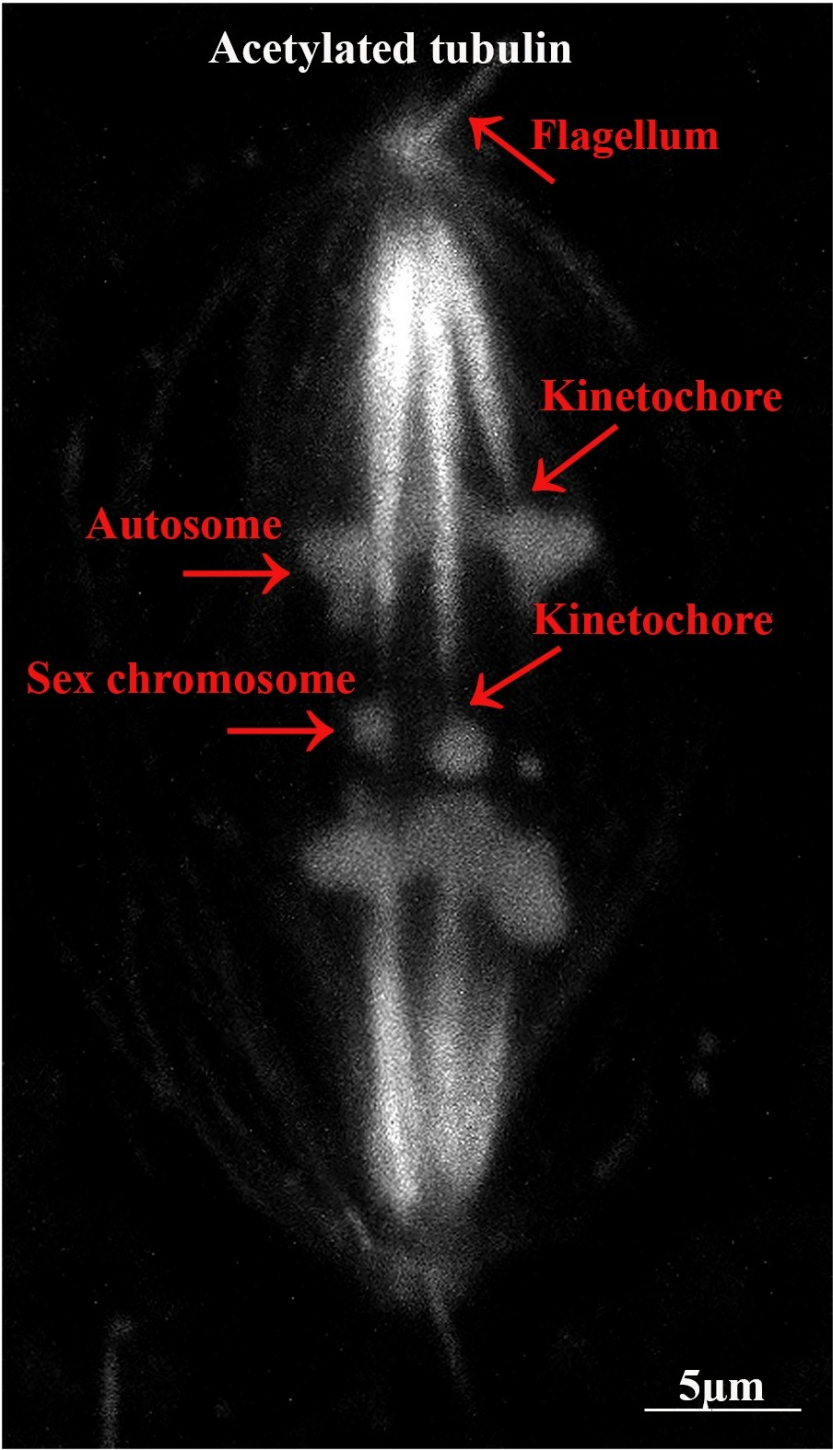
Autosomes versus sex chromosomes in crane fly spermatocyte. A control cell and a normal mid-anaphase stained for acetylated tubulin. During anaphase autosomes start moving toward the poles while the sex chromosomes remain at the equatorial plane until the autosomes reach to the poles.

**Fig 2:**
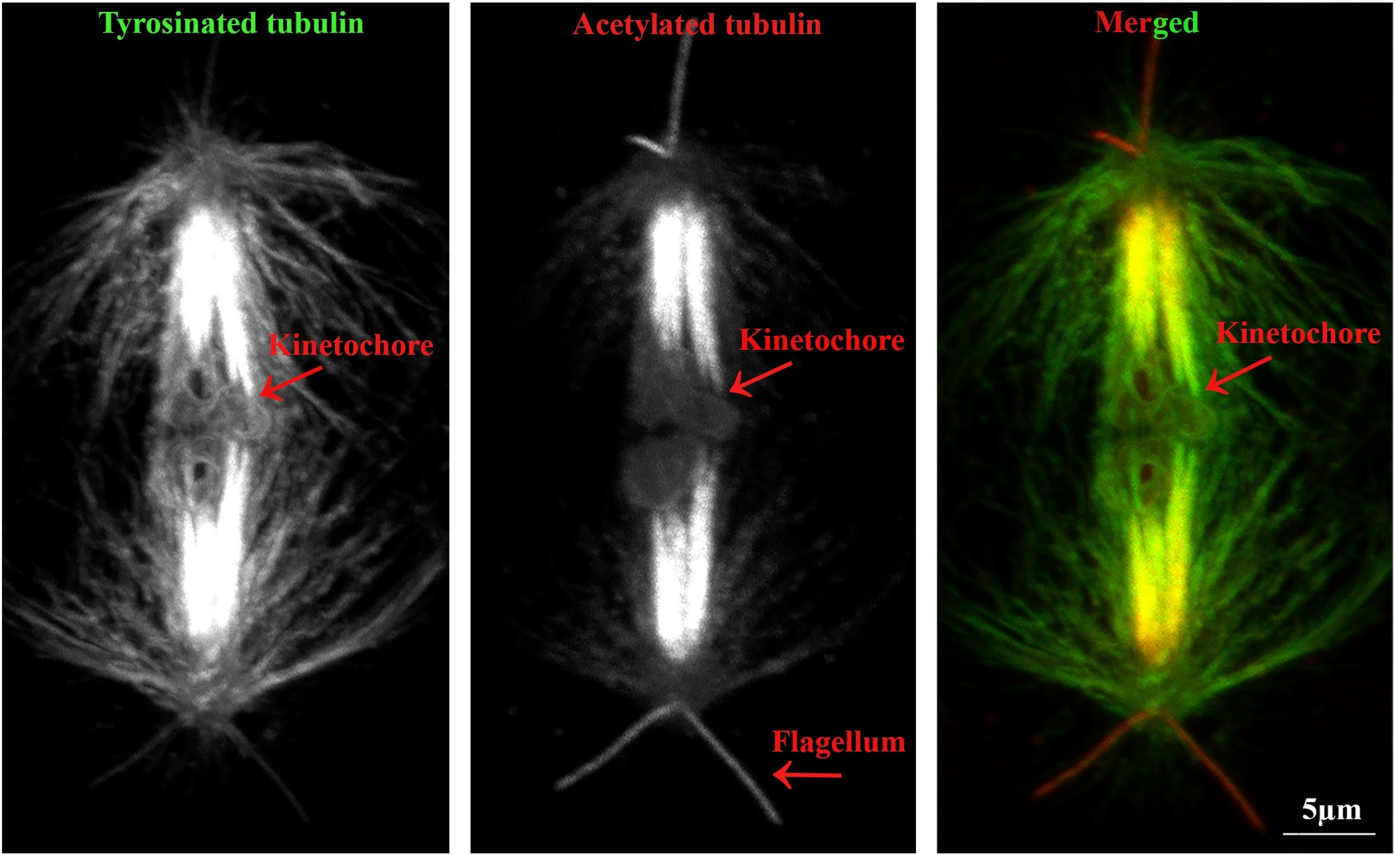
Crane fly spermatocyte stained for tyrosinated and acetylated-α-tubulin. (An early anaphase spindle stained for tyrosinated tubulin which contains astral, interpolar and kinetochore microtubules. However, in acetylated-α-tubulin channel only kinetochore microtubules and flagella are strongly stained. The merged picture which shows both tyrosinated-α-tubulin (in green) and acetylated-α-tubulin (in red) is visualized by dual channel imaging with confocal microscopy. The merged picture shows that microtubules are highly acetylated at the poles (more red color) and there is a lack of acetylation right at the kinetochore (no red color).

Crane-fly primary spermatocytes have three autosomal bivalents and two sex-chromosome univalents [18]. The autosomal chromosome kinetochores are monotelic: each half-bivalent is attached to one of the poles only, and the two half-bivalents (that make up a single bivalent) are attached to opposite poles. The sex chromosome kinetochores are amphitelic: each univalent has kinetochore fibers to both poles. During anaphase of the first meiotic division, autosomal chromosomes (half-bivalents) move from the equator to the opposite poles, but the sex chromosomes remain at the equator and do not move poleward until the autosomes reach the poles (Fig 1) [18,19]. Kinetochore microtubules, those that extend between chromosome and pole, need to be depolymerised during anaphase; they are acetylated in a variety of eukaryote cells [11,20–26]. In primary crane-fly spermatocytes, out of all the cytoplasmic and spindle microtubules present, only the kinetochore microtubules (and those of the two flagella at each pole) seem to be acetylated [11,27]. This was determined by labelling metaphase and anaphase spermatocytes with antibodies to both acetylated-α-tubulin and tyrosinated-α-tubulin: only the kinetochore microtubules and flagella were stained by the acetylated α-tubulin antibodies. In addition, in the acetylated microtubules there was a ‘gap’ in staining at the kinetochore where staining only began to be strong a few micrometers from the kinetochore (Fig 2). Conversely, tyrosinated-α-tubulin stained evenly from pole to kinetochore. The ‘gap’ in acetylated tubulin near the kinetochore is because of the time lag between microtubule polymerization at the kinetochore and subsequent acetylation as the tubulin subunits move (flux) toward the pole. The gap in acetylation near the kinetochore was present both in metaphase and anaphase cells, which suggested to Wilson and Forer (1997) that depolymerization of the kinetochore microtubules is at the pole during anaphase, a conclusion that was directly verified by following injected fluorescent tubulin during anaphase [27,28].

To test the hypothesis that deacetylation must occur in order for acetylated kinetochore microtubules to be depolymerized during normal anaphase, we used two different HDAC6 inhibitors to assess chromosome movement. In some cases, chromosomes slowed down after drug perfusion, while in others, they stopped and started again spontaneously, and some that stopped then resumed normal anaphase movements after washing out the drug. Our results support the hypothesis that acetylated kinetochore microtubules must be deacetylated in order for microtubules to be depolymerized during normal anaphase.

## Materials and methods

All experiments were conducted at room temperature (25°C) as movement speeds are temperature dependent [29].

### Living cell preparation and cell treatment

Our laboratory stock of *Nephrotoma suturalis* (Loew) was reared essentially as described in Forer (1982) [18]. We used testes of fourth instar larvae to obtain primary spermatocytes, using methods described previously [30]. Briefly, testes were removed from larvae under halocarbon oil (to prevent evaporation). The testes were placed in Ringers solution (0.13M NaCl, 5mM KCl, 1mM CaCl_2_, 0.02 M Na_2_HPO_4_-KH_2_PO_4_ buffer pH: 6.8) which contained fibrinogen. After the cells were spread out, thrombin was added to form a clot. Then the coverslip and cells were attached to a perfusion chamber and the chamber was filled with insect Ringers solution in order to bathe the cells in isotonic medium and prevent the cells from drying out. For inhibiting deacetylation by HDAC6 we added to the Ringers solution either Tubacin (Cayman Chemicals, Ann Arbor, MI, USA or Sigma-Aldrich, St. Louis, MO, USA) or TSA (TSA; Cayman Chemicals, Ann Arbor, MI, USA). The IC50 for Tubacin inhibiting HDAC6 is 4nM [31] and that for TSA is 2.5nM [32]. Tubacin is a specific inhibitor for HDAC6 that prevents deacetylation of α-tubulin without any effect on histone acetylation [16]. The final concentrations of Tubacin we added to the cells were 4 nM, 35 nM, 100 nM, 200 nM, 400 nM, 600 nM and 1.4μM, and of TSA was 2.5 nM. The drugs were stored at - 20°C in dimethyl sulfoxide (DMSO) and diluted from concentrated stock solutions; for all treatments the stock solutions were diluted 1:1000 into Ringer solution before adding to the cells. Previous control experiments have shown that 0.1% DMSO has no effects on chromosome movement and cell division in crane-fly spermatocytes [33].

### Measurement of chromosome movement

Living cells were observed using a 100x oil-immersion Nikon phase-contrast objective lens, NA = 1.3, and images were recorded in real time on DVDs. The recorded images were converted into avi files using VirtualDub2 Freeware. Using WinImage, an in-house program, we measured either distances between separating kinetochores during anaphase chromosome movement or distances between kinetochores and a fixed point at the pole. The intervals between measured images were 4 or 10 seconds. The results were graphed using the commercial program SlideWrite.

All data are presented as boxplots showing the median, interquartile ranges (IQR), and 1.5x the IQR with individual data points. The creation of figures and statistical analysis were conducted using Rstudio 2023.6.1.524 [34]. Data were assessed for normality by visual inspections of quantile-quantile plots of model residuals while homogeneity of variance was assessed by a visual inspection of the plots of model residuals against fitted values. Differences in chromosome movement were assessed using linear models. Main effects were determined using the “Anova” function. When necessary, estimated marginal means were calculated using the “emmeans” package to examine pairwise post-hoc comparisons [35]. An alpha level of 0.05 was used throughout.

### Immunofluorescence

To study immunofluorescence, preparations of drug-treated cells or of control cells were lysed by immersing them for ≥10 minutes in a lysis buffer (100 mM piperazineN, N-bis (2-ethanesulfonic acid) (PIPES); 10mM EGTA; 5mM MgSO_4_; 5% DMSO; 1%Nonidet P-40; pH 6.9). The lysed cells then were fixed by immersion for ≥5 minutes in 0.25% glutaraldehyde in phosphate-buffered saline (PBS) and then were rinsed 3 times for 5 minutes each in PBS and were put into 0.05M glycine for 10 minutes to neutralize any free aldehyde groups. They were rinsed again in PBS, 3 times, each for 5 minutes. The final fixed coverslip was stored in 1:1 PBS-glycerol at 4°C until it was processed further for immunofluorescent staining. For immunofluorescence, the cells were stained both for tyrosinated-α-tubulin and for acetylated-α-tubulin. The staining procedures were at room temperature and in subdued lighting in order to prevent light inactivation of the fluorochromes. First the coverslips were floated cell side down on PBS (three times for 15 minutes each) to removed PBS/glycerol. After removing glycerol, the coverslips were rinsed once with PBS that contained 0.1% Triton-X in order to facilitate spreading of the antibodies. Then the cells were incubated with the two primary antibodies for an hour. After primary antibody incubation, the cells were washed by PBS three times each for 5 minutes, and again PBS wash containing 0.1% Triton X. Then the cells were incubated with the two secondary antibodies, for one hour. Then the slides were rinsed with PBS and placed on a slide in a Mowiol-containing solution [36], to which we added 0.2 g/litre of the anti-fading agent, paraphenylene-diamine. The two primary antibodies were: (1) YL1/2, a monoclonal rat IgG2a against tyrosinated-α-tubulin (α-TYR) (Serotec Limited, Oxford, England) diluted 1:500 in PBS, and (2) 6–11 B-1, a monoclonal mouse IgG2bk against acetylated tubulin (Sigma) diluted 1:20 in PBS. The two secondary antibodies were: (1) Alexa 488 Goat anti-rat IgG (diluted 1:100 in PBS), from Thermo-Fisher, cross-adsorbed against mouse, and (2) Alexa 568 Goat anti-mouse IgG (diluted 1:100 in PBS), from Thermo-Fisher, cross-adsorbed against rat. The slides were left in the dark in the fume hood until the Mowiol hardened.

Cells were then observed using a Zeiss LSM confocal microscope. We used the argon laser at 488nm and HeNe laser at 555nm for exciting the fluorophores and we imaged the preparations with a Plan Apo 63x oil immersion objective (NA: 1.4). To see the chromosomes and kinetochores, we added another channel for Differential Interference Contrast (DIC). Each image was taken as a series of optical sections spaced 0.38μm apart (Z-series). All the images were taken with Zen black edition software and further analysis was done using Image J (FIJI).

### Intensity analysis and “gap” measurement

**Fig 3:**
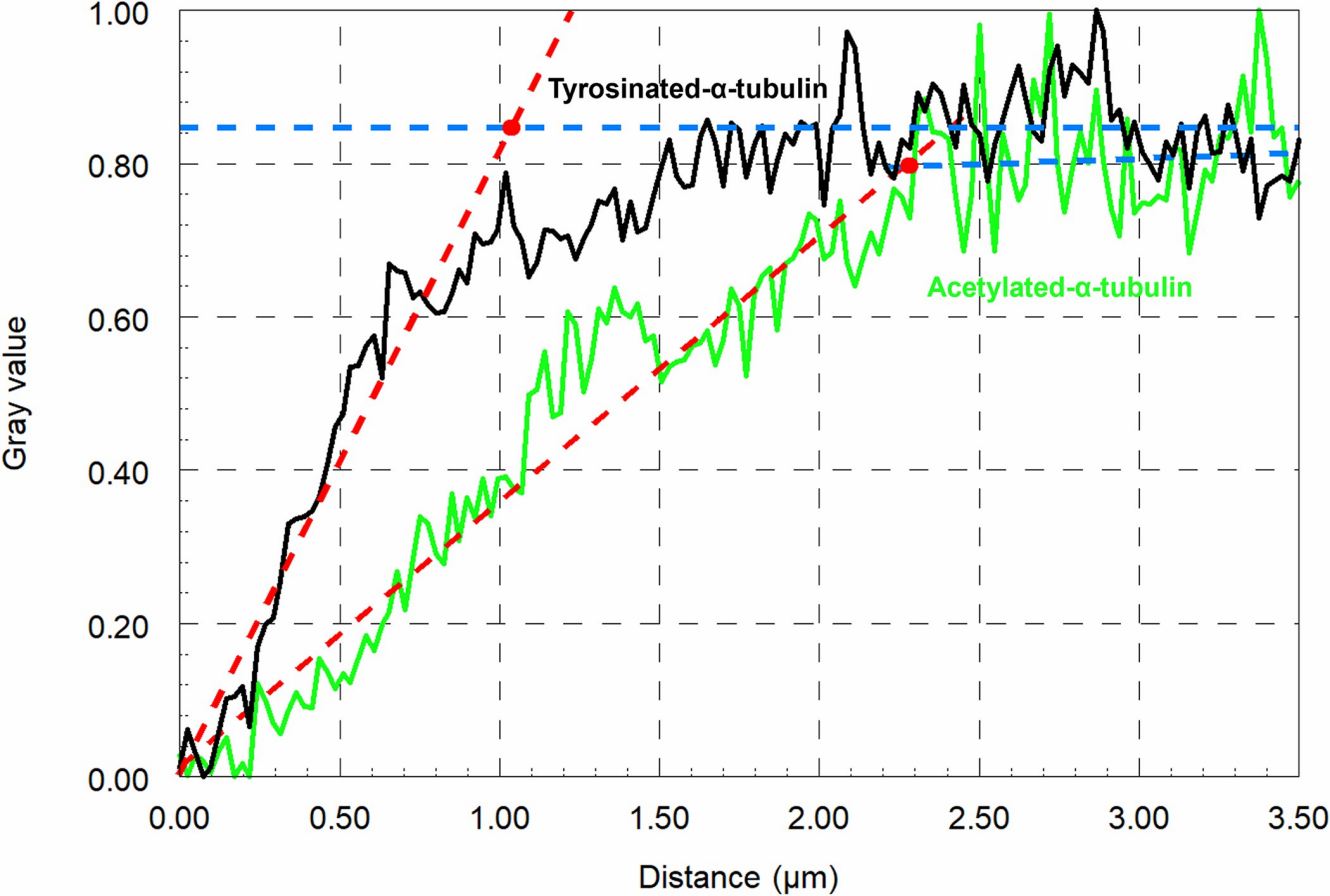
Difference between intensity profiles for tyrosinated-α-tubulin and acetylated-α-tubulin. In both graphs the blue line is the best fit for the maximum staining intensity values, and the red line is the best fit for the regions of sharp slope which represents the increase in staining from the kinetochore (at 0.00). Both red and blue lines are determined by SlideWrite. The intersection of the two lines (Red dot) represents the position from the kinetochore of maximum staining intensity of tyrosinated α-tubulin and acetylated α-tubulin. The difference between the maximum intensity of tyrosinated α-tubulin and acetylated α-tubulin represents the gap of staining (acetylation) at the kinetochore.

To measure the lengths of the kinetochore microtubules near the kinetochore that did not stain strongly with acetylated tubulin, we compared the fluorescence intensity profiles in the tyrosinated-α-tubulin and acetylated-α-tubulin channels. DIC images were used to determine the positions of kinetochores; to obtain the staining intensities of α-acetylated-tubulin and tyrosinated-α-tubulin we placed a line on the kinetochore fibres using Image J/Fiji. After we visually and with the help of DIC images determined that both lines were accurately placed, we obtained plots of fluorescence intensity *versus* position along the line using Image J/Fiji. Sample intensity profiles for both tyrosinated-α-tubulin and acetylated-α-tubulin channels are shown in Fig 3. The 0.00 position on each graph is at the kinetochore as seen in the DIC image and continues toward the pole. The scan is in exactly the same position in both images, along the length of the kinetochore fiber. As seen in Fig 3 (black line), the intensity of tyrosinated α-tubulin labelling increased sharply from the kinetochore towards the pole and reached the highest intensity at ~1 μm from the zero point. The acetylated α-tubulin increased less sharply and reached its highest intensity at a distance ~2.5 μm from the kinetochore (Fig 3, green line). Following Wilson et al. 1994, who quantified the difference between acetylated tubulin and tyrosinated tubulin staining at kinetochores, we call the difference between the maximum intensity of tyrosinated-α-tubulin and the maximum intensity of α-acetylated-tubulin the “gap” in kinetochore fiber acetylation [37].

## Results

### Effects of tubacin on anaphase chromosome movement

**Fig 4:**
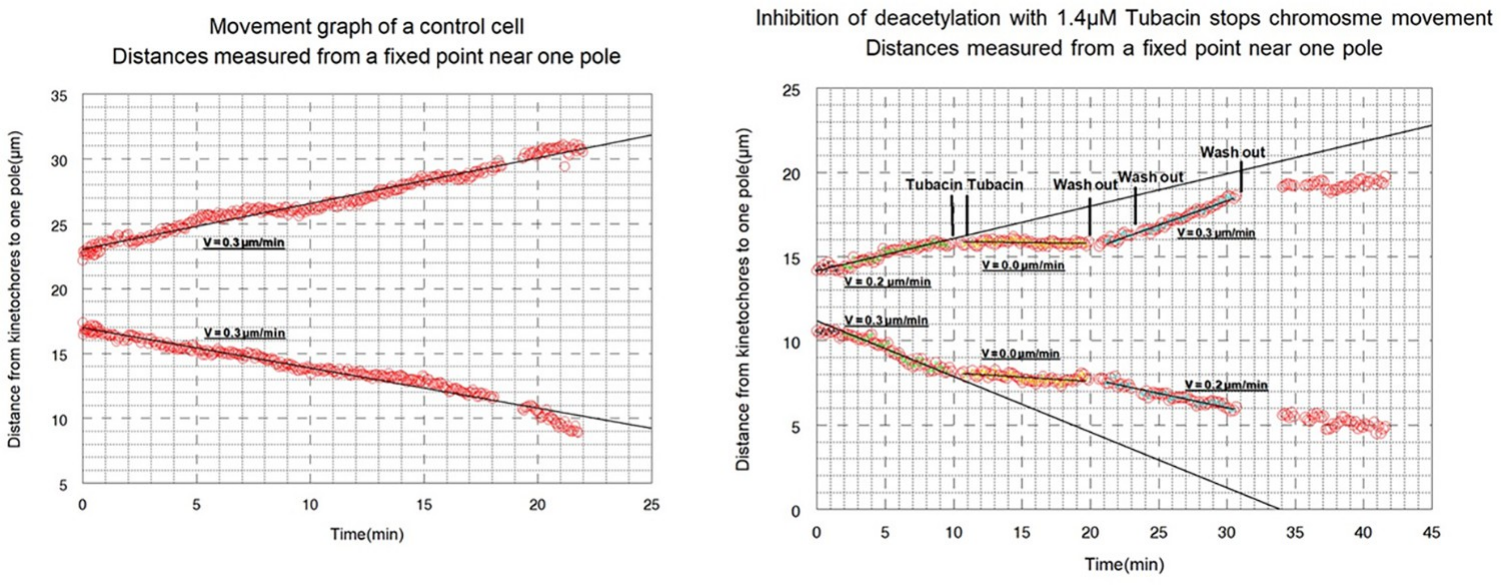
Effect of Tubacin on chromosome anaphase movement. (Control cell) Anaphase movement of one pair of chromosomes in a cell in Ringers solution without any drug treatment. The graph starts shortly after the beginning of anaphase. The circles represent the positions of the two kinetochores moving to the upper and lower poles and the slopes of the lines represent the velocities of movement (0.3μm/min for each half-bivalent). (Drug treated cell) The spermatocyte was treated with 1.4μM Tubacin. The slopes of the black lines indicate the speeds of the chromosomes before drug perfusion (0.2μm/min, 0.3μm/min), the speeds of the chromosomes after drug perfusion (0.0μm/min, 0.0μm/min), and the speeds of the chromosomes after washing out the drug with Ringers solution (0.3μm/min, 0.2μm/min). Both partner chromosomes stopped after drug treatment and restarted moving after wash-out.

**Fig 5:**
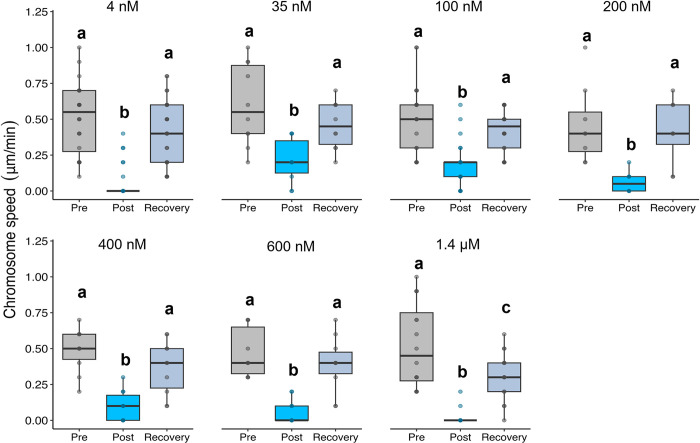
Chromosome movement velocity (μm/min) at various concentrations of tubacin treatment. Chromosome movement velocity (μm/min) before the treatment is added (Pre; grey boxes), after the addition of tubacin (Post; blue boxes), and after washing the treatment out (Recovery; light blue boxes). Data are presented as boxplots where the center line represents the median, the length of the box extends to the IQR, and the whiskers extend to 1.5x IQR with individual data points shown. Boxes not sharing a letter were found to be significantly different (p < 0.05) as determined by linear models with estimated marginal means used in the post-hoc analysis.

We first quantified chromosome movement velocities to their poles after various concentrations of tubacin treatment. The average speed of poleward motion for single half-bivalents pre-treatment was 0.5 μm/min ± 0.24 (SD), (n = 53 pairs; Fig 4). For each of 106 chromosomes in 53 pairs, both half bivalents of each pair moved with the approximately same speed (Fig 4). Tubacin significantly impaired chromosome speed regardless of concentration (0.1 μm/min ± 0.13; p < 0.001) with speeds to each pole being either 0 μm/min (n = 28 pairs, chromosomes stopped) or 0.18 μm/min ± 0.1 (n = 18 pairs, chromosomes slowed down). In some cases, chromosomes kept moving poleward after drug treatment without any effects in lower concentrations (lower than 400 nM, n = 7 pairs). The effect of inhibitor was reversible, chromosome pairs moved toward the poles after the treatment was washed out (0.4 μm/min. ± 0.22; p < 0.001). Interestingly, it was observed in some chromosome pairs in the lower Tubacin concentrations that movement would resume even before the drug was washed out, while this was not observed in the higher concentrations. Following the Ringers wash, all concentrations showed a recovery of speed back to pre-treatment levels (p > 0.05) except the 1.4 μM treatment (our highest concentration), for which a full recovery was not observed (0.3 μm/min. ± 0.16; p = 0.004) (Fig 5). Interestingly, some chromosomes started moving backward toward the equator after cells were treated with drugs, probably because the tethers between separating telomeres exert backward forces on them [38]. The backward movements had average speeds of 0.15 μm/min ± 0.07 in n = 18 pairs. Because the tethers were likely forcing this movement, backward movements were excluded from the chromosome speed analysis.

### Trichostatin A (TSA)

**Fig 6:**
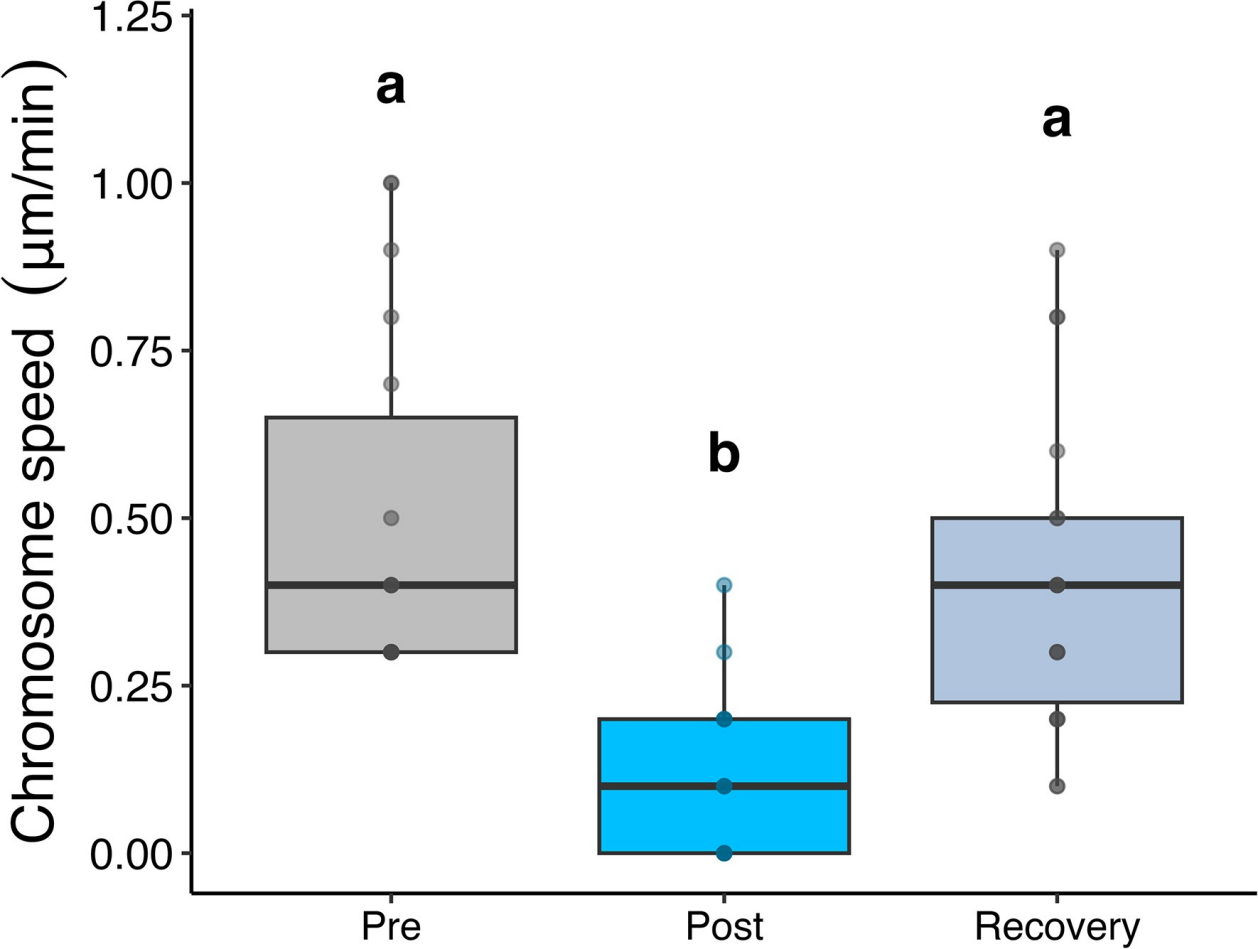
Chromosome movement velocity (μm/min) at 2.5nM TSA treatment. Chromosome movement velocity (μm/min) before the treatment is added (Pre; grey boxes), after the addition of TSA (Post; blue boxes), and after washing the treatment out (Recovery; light blue boxes). Data are presented as boxplots where the center line represents the median, the length of the box extends to the IQR, and the whiskers extend to 1.5x IQR with individual data points shown. Boxes not sharing a letter were found to be significantly different (p < 0.05) as determined by linear models with estimated marginal means used in the post-hoc analysis.

We examined another HDAC6 inhibitor, Trichostatin A (TSA), to investigate whether the effects of Tubacin actually are because it acted on HDAC6 or rather because Tubacin acted on a different component and the observed effect was a drug side effect. TSA interacts with a different part of the HDAC6 active site than Tubacin [39]. We treated five cells with 2.5 nM TSA, and we obtained data on 9 chromosome pairs. The average speed of poleward half-bivalent movement before drug perfusion was 0.5 μm/min. TSA treatment significantly decreased chromosome speed (0.11 μm/min. ± 0.12; *p* < 0.001) and all effects were recoverable when compared to pre-drug conditions (0.41 μm/min. ± 0.24; *p* = 0.25) (Fig 6).

### Immunostaining of acetylated tubulin

We examined acetylated microtubules in treated cells to see if blocking deacetylation caused changes in acetylated kinetochore microtubules.

The only microtubules that are acetylated in crane-fly primary spermatocytes are kinetochore microtubules. The only other components stained are the two acetylated flagella at each spindle pole (Figs 1 and 2). By quantifying the staining intensities of flagella and kinetochore fibers in the same cell we could estimate how many acetylated microtubules were present in the kinetochore fiber. We scanned as many flagella per control cell as we could see clearly and scanned kinetochore fibers in the same cells. Since flagella have 9 doublet microtubules +2 central singlet microtubules = 20 microtubules, we used the flagellar fluorescence as a standard to estimate the number of acetylated microtubules in the kinetochore microtubule bundle. We took ratios of maximum intensities in the kinetochore fibers divided by maximum intensities in a flagellum in the same cell. We multiplied the staining intensity ratios by 20 to estimate the number of acetylated microtubules in the kinetochore fiber. For twenty-two such ratios, the estimated number of acetylated microtubules in the kinetochore fiber was 42 ± 12.4 (SD), with range 31 to 68 (except for one kinetochore fiber that had 25).

**Fig 7:**
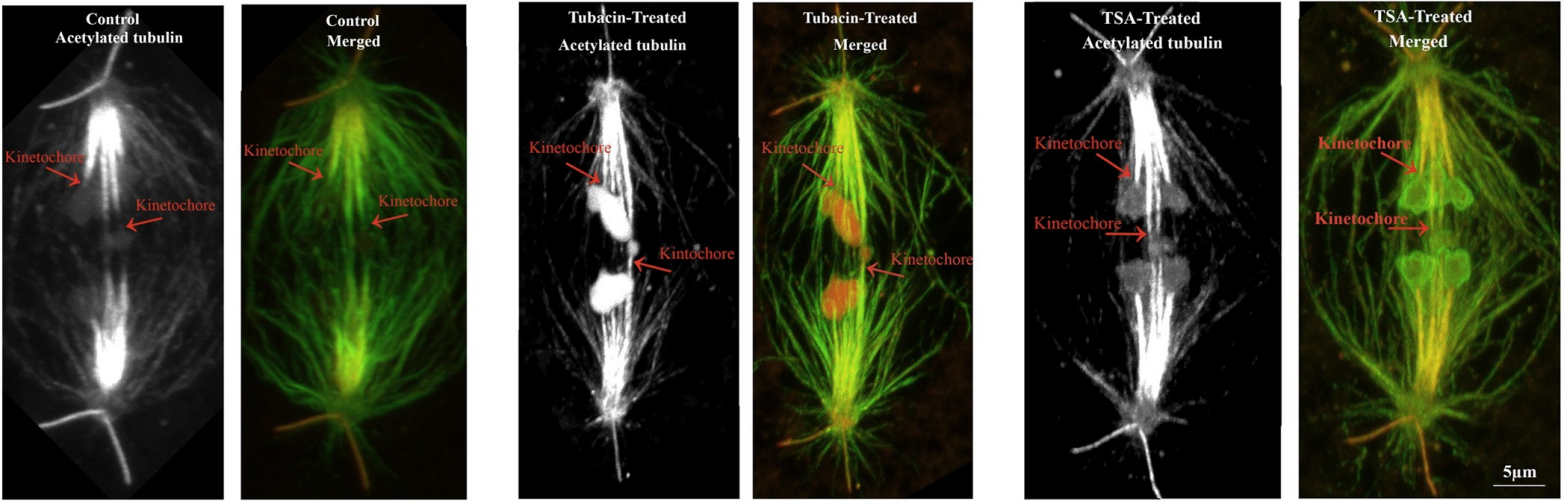
Confocal Z-series of control cell versus treated-cells stained for tyrosinated-α-tubulin and acetylated-α-tubulin. (Control) A cellin mid-anaphase with acetylated tubulin channel and a merged image of both tyrosinated and acetylated channels, (Tubacin treated) Cell in mid-anaphase treated with 4nM Tubacin and (TSA treated) Cell in a same stage of anaphase as (Control) and (Tubacin treated) treated with 2.5nM TSA. As seen in the drug treated cells, the acetylated-α-tubulin is evenly stained from pole to the kinetochore which means the ‘gap’ at the kinetochore no longer exists after inhibition of HDAC6.

**Fig 8:**
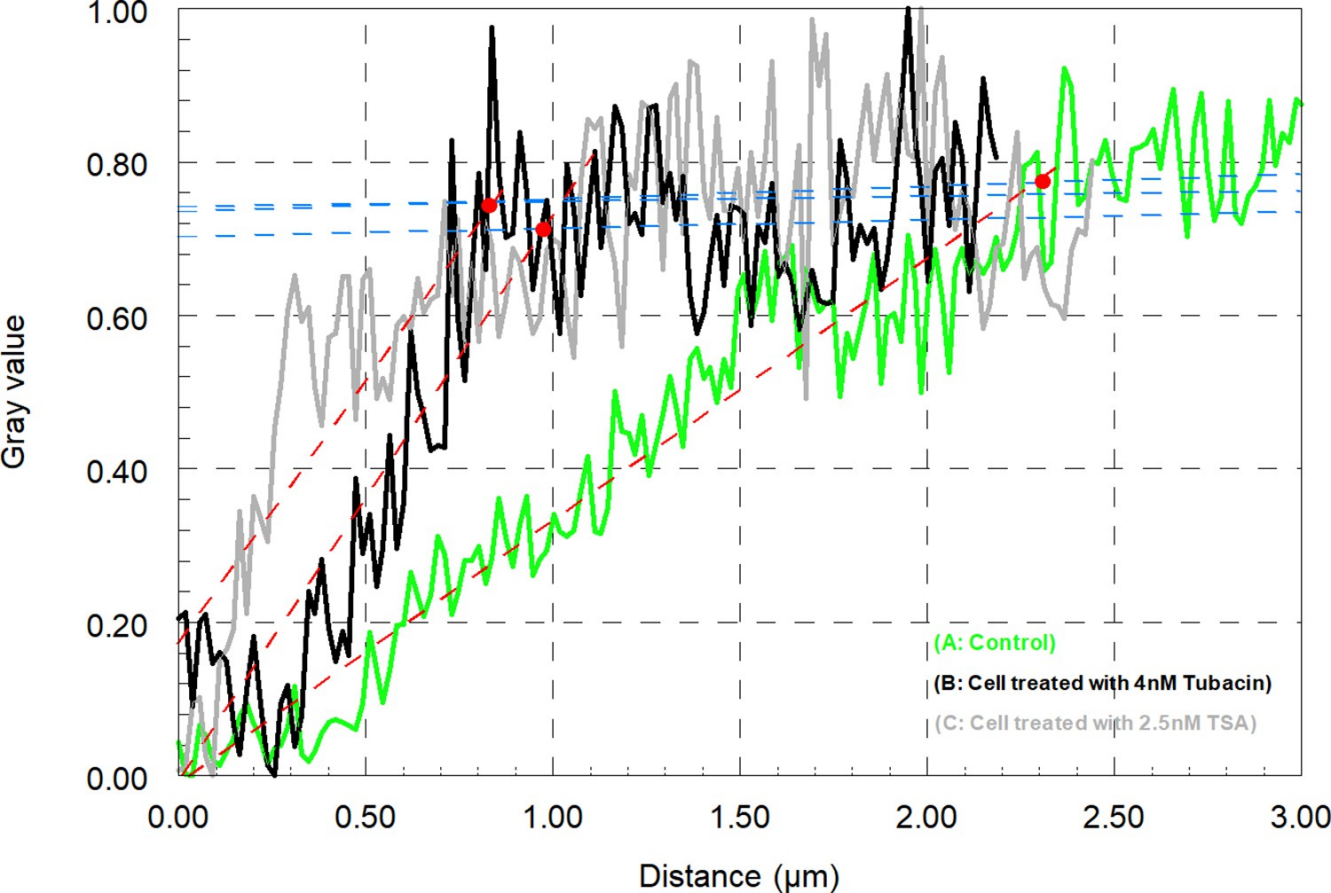
Difference in acetylated-α-tubulin intensity profiles between control and drug-treated cells. The blue line in each graph indicates our estimated maximum staining intensity (the best fit line for maximum intensity), while the red line (the best fit line for slope regions) shows the increase in staining from the kinetochore (at 0.00). Both red and blue lines are determined by SlideWrite. (graph A, green line): The fiber labeled for acetylated α-tubulin in a control cell. The intersection of the red and blue lines, marked by a red dot, represents the position from the kinetochore of maximum staining intensity of acetylated tubulin. In the control cell (A), the staining intensity peaks at approximately 2.5 μm, indicating a gap in acetylation. (graphs B and C, black and gray lines respectively): The fibers labeled for acetylated α-tubulin in drug-treated cells with Tubacin and TSA. In both Tubacin and TSA-treated cells, the staining intensity peaks at less than 1 μm, indicating that the acetylation gap disappears following HDAC6 inhibition.

We were interested in studying acetylated tubulin in kinetochore microtubules after treatment with Tubacin and/or Trichostatin A to see if this might give us clues about the consequences of blocking (or slowing) microtubule deacetylation. In control cells we saw gaps in staining for acetylated microtubules near the kinetochores in both metaphase and anaphase. We quantified the “gap” in 27 control cells (12 cells in different stages of anaphase and 15 cells in metaphase) by measuring the intensity of labelled spindle fiber as shown in Fig 3. We plotted the intensities of acetylated α-tubulin as well as tyrosinated-α-tubulin. The acetylated gap at the kinetochore persists in control cells, in both anaphase and metaphase, consistent with the data from LaFountain et al. (2004) from injection of fluorescently labelled tubulin [28]. The treated cells that we studied were kept in the drugs (Tubacin and TSA) for at least 10 minutes before lysing them for the immunostaining procedure. We observed 17 cells treated with Tubacin or TSA, both in metaphase and anaphase. In all treated cells there was no gap in staining of acetylated tubulin at the kinetochore (Figs 7 and 8). Inhibiting deacetylation caused by HDAC6 stops or slows chromosome movement, which may arise because tubulin flux and treadmilling to the pole were stopped. We interpret this to mean that new tubulin subunits that were added to the kinetochore became acetylated *in situ*, without fluxing to the pole, presumably because acetylated microtubules are not able to depolymerize at the pole.

There was one other difference between kinetochore microtubules in control *versus* drug-treated cells that we noted. The kinetochore microtubule bundles seen after inhibition of HDAC6 seemed to be visibly thinner than those in the control cells, as seen clearly in Fig 7 compared with Figs 1 and 2. We have not quantified or studied this in detail, but it may represent some change in kinetochore spindle fiber structure.

## Discussion

Our data shows that chromosome movements can be affected by inhibiting HDAC6 with Tubacin and that the effects were dose-dependent and reversible. We do not think this is a side effect of one particular drug because we got similar effects with two different drugs that act on different regions of HDAC6. The main conclusions from our experiments are that inhibiting deacetylation of kinetochore microtubules during anaphase stops or slows chromosome anaphase movements, and we assume that inhibiting deacetylation blocks the flux of tubulin subunits incorporated at the kinetochores, which can be evaluated in future experiments.

Kinetochore microtubules, which are acetylated during cell division, must depolymerize at the poles in order for the chromosomes to move during anaphase. Meanwhile, kinetochore microtubules polymerize at the kinetochore, meaning they are dynamic during cell division [28]. We assume that the flux and treadmill-like walking of tubulin subunits in kinetochore microtubules causes a gap in acetylation at the kinetochore due to the time lag between polymerisation and acetylation (Figs 2 and 3). Following the addition of Tubacin, which inhibits HDAC6 and therefore the deacetylation of microtubules, we observed a significant reduction in chromosome speed which we attribute to the immediate blocking of kinetochore microtubule deacetylation. Therefore, it seems to us that blocking deacetylation inhibits the depolymerization of kinetochore microtubules at the pole, which in turn alters chromosome movements. However, the exact effect likely depends on how efficient the block is and how many acetylated microtubules remain following drug treatment. The hypothesis is that the gap in acetylation at the kinetochore disappears as the flux is inhibited, so newly incorporated tubulin units sit and do not move poleward; after some time being stationary, they are for all intents and purposes stable and become acetylated (Figs 7 and 8).

Based on published data we know that HDAC6 is associated with microtubules in the cytoplasm and localizes with microtubule motor proteins [15]. Our interpretation assumes that HDAC6 deacetylates kinetochore microtubules at the pole, so that the tubulins can be deacetylated in order to be depolymerized. Future work could investigate this by studying whether HDAC6 is indeed present at spindle poles.

Our interpretation also assumes that most kinetochore microtubules are acetylated. We estimated from staining intensities that there were ≈42 acetylated microtubules per kinetochore fiber (range 31–68). For confirmation of our estimates, we compared these values with the total numbers of microtubules in kinetochore fibers determined previously by others using electron microscopy or quantitative polarisation microscopy. The numbers obtained from fluorescent ratios are in the range of the numbers of kinetochore fibre microtubules estimated from electron microscopy and from quantitative polarisation microscopy, which range from 40 to 90 microtubules per kinetochore fiber (Table 1). Thus, it seems that most, if not all, of the kinetochore microtubules are acetylated.

**Table 1:** Numbers of kinetochore fiber microtubules in Nephrotoma primary spermatocytes.

Method	Microtubules per kinetochore fiber	Reference
**Acetylated-Tubulin staining**	≈ 42	This article
**Electron microscopy**	70–90	LaFountain et al 1976
	59	Fuge* 1980
	40–50	Fuge* 1973
**Quantitative polarisation microscopy**	29–73	LaFountain & Oldenburg 2014

*: Fuge studied Nephrotoma ferruginea, whereas we and the others studied N. suturalis.

Various data show that in mitotic spindles kinetochore microtubules are selectively acetylated. Wilson and Forer (1989) found that in crane-fly spermatocytes during different stages of the cell division, kinetochore microtubules are highly acetylated and the other spindle microtubules were not acetylated at all [11]. Similarly in flea beetle spermatocytes [25]. Others showed that in anaphase the kinetochore microtubules are most highly acetylated at the poles and the older microtubules appear to be acetylated and more stable than the newly added microtubules [11,20,21,23–25].

Several studies showed that tubulin becomes deacetylated specifically via HDAC6 [40]. Tubacin and TSA inhibit HDAC6 and induce significant microtubule hyperacetylation [41]. Tubacin greatly affects the velocities of microtubule growth and shortening in B16F1 melanoma cells [42], consistent with our interpretation of Tubacin´s effect on kinetochore microtubules.

Our data suggest that inhibiting HDAC6 affects chromosome movement to the pole, in a reversible manner at low concentrations of the drugs. This supports the hypothesis there is a link between microtubule deacetylation and depolymerization. In anaphase crane-fly spermatocytes kinetochore microtubule depolymerization occurs at the pole [11,28]. Thus, by inhibiting HDAC6 and thereby inhibiting deacetylation of kinetochore microtubules, the microtubules remain stable, the kinetochore microtubules stop depolymerization, and chromosome movement stops or slows down, depending on inhibitor concentrations.

The effects of deacetylation inhibitors were on chromosome pairs, not individual chromosomes, and the effects were different in chromosome pairs in the same cell. That the two partners in a separating pair are affected the same way most likely is due to the elastic mitotic tethers between the partners [38], since other data point to tethers coordinating the movements of partner chromosomes during anaphase [43,44]. Why do different pairs in the cell sometimes respond differently? We considered one possible explanation for this phenomenon. There are several mechanisms in a cell for tubulin deacetylation. Previous studies showed that besides HDAC6, which plays a crucial role in α-tubulin deacetylation, SIRT2 acts as a deacetylase for lysine-40 of α-tubulin both *in vitro* and *in vivo*. Brian et al., (2003) inhibited SIRT2 by siRNA which resulted in tubulin hyperacetylation [45]. Interestingly, the tubulin deacetylase activity of SIRT2 is not affected by HDAC6 inhibitors, but it is inhibited by the drug ‘nicotinamide’ which is a specific inhibitor for class III HDACs [46]. Despite the fact that previous observations indicated that inhibition of either HDAC6 or SIRT2 is adequate to induce hyperacetylation of tubulins, our hypothesis suggests that in some cells when we inhibit HDAC6, there might be redundancy of enzymes such that the kinetochore microtubules of some half-bivalents might use SIRT2 for deacetylation and continue moving normally because SIRT2 deacetylates enough of the kinetochore microtubules to allow poleward movement. This hypothesis also can explain half-bivalents that behaved differently in a same spermatocyte, for example where one pair of half-bivalents wasn’t affected by the drug and moved with a normal speed to the pole and the other pair slowed down significantly after drug perfusion. The differences between pairs might arise because different pairs use different mechanisms for deacetylation or may switch to a different mechanism. Thus, it is possible that the kinetochore microtubules of one pair become deacetylated by HDAC6, and the other becomes deacetylated by SIRT2. That might explain why when we inhibit HDAC6, some half-bivalents stop, and some others continue moving.

In some cells treated with inhibitors, we observed that some half-bivalents moved backward after they stopped moving poleward. The most likely explanation is that the backward movements are due to elastic ‘tethers’ that extend between separating anaphase chromosomes. Various experiments have shown that tethers are physical elastic connections between segregating partners’ telomeres during anaphase [38]. If poleward forces decrease due to inhibition of deacetylation, this blocks the poleward force on the kinetochores so when tethers exert backward force on chromosomes arms, the half-bivalents could move backward toward the equatorial plane, as they do after other treatments [47].

In sum, we have shown that inhibiting deacetylation of kinetochore MTs by adding inhibitors during anaphase slows and stops chromosome movement. We suggested that this was because the acetylated microtubules could not be depolymerised at the poles and therefore chromosome movement stopped or slowed. Supporting this hypothesis is that, as shown by staining of acetylated tubulin, the inhibition of movement is accompanied by stopping tubulin incorporation at the kinetochore that in these cells normally takes place during anaphase. However, direct evidence for altered tubulin incorporation at kinetochore requires further investigation analysis of microtubule flux under these conditions.

## References

[1] Pickett-Heaps J, Forer A, Spurck T. Rethinking anaphase: where “Pac-Man” fails and why a role for the spindle matrix is likely. Protoplasma. 1996;192:1–10.

[2] Maddox P, Straight A, Coughlin P, Mitchison TJ, Salmon ED. Direct observation of microtubule dynamics at kinetochores in Xenopus extract spindles: implications for spindle mechanics. The Journal of cell biology. 2003;162(3):377. doi: 10.1083/jcb.200301088 12900391 PMC2172681

[3] Forer A, Wilson P. A model for chromosome movement during mitosis. Protoplasma. 1994;179:95–105.

[4] Forer A, Spurck T, Pickett-Heaps J. Actin and myosin inhibitors block elongation of kinetochore fibre stubs in metaphase crane-fly spermatocytes. Protoplasma. 2007;232:79–85. doi: 10.1007/s00709-007-0265-8 18094930

[5] Rasamizafy SF, Delsert C, Rabeharivelo G, Cau J, Morin N, Van Dijk J. Mitotic acetylation of microtubules promotes centrosomal PLK1 recruitment and is required to maintain bipolar spindle homeostasis. Cells. 2021;10(8):1859. doi: 10.3390/cells10081859 34440628 PMC8394630

[6] Carmona B, Marinho HS, Matos CL, Nolasco S, Soares H. Tubulin post-translational modifications: the elusive roles of acetylation. Biology. 2023;12(4):561. doi: 10.3390/biology12040561 37106761 PMC10136095

[7] Zadra I, Jimenez-Delgado S, Anglada-Girotto M, Segura-Morales C, Compton ZJ, Janke C, et al. Chromosome segregation fidelity requires microtubule polyglutamylation by the cancer downregulated enzyme TTLL11. Nature Communications. 2022;13(1):7147. doi: 10.1038/s41467-022-34909-y 36414642 PMC9681853

[8] Wattanathamsan O, Pongrakhananon V. Post-translational modifications of tubulin: their role in cancers and the regulation of signaling molecules. Cancer Gene Ther. 2023;30(4):521–8. doi: 10.1038/s41417-021-00396-4 34671113

[9] Song Y, Brady ST. Post-translational modifications of tubulin: pathways to functional diversity of microtubules. Trends in cell biology. 2015;25(3):125–36. doi: 10.1016/j.tcb.2014.10.004 25468068 PMC4344850

[10] Nekooki-Machida Y, Hagiwara H. Role of tubulin acetylation in cellular functions and diseases. Medical molecular morphology. 2020;53(4):191–7. doi: 10.1007/s00795-020-00260-8 32632910

[11] Wilson P, Forer A. Acetylated α‐tubulin in spermatogenic cells of the crane fly Nephrotoma suturalis: Kinetochore microtubules are selectively acetylated. Cell motility and the cytoskeleton. 1989;14(2):237–50.

[12] Burbank KS, Mitchison TJ. Microtubule dynamic instability. Current Biology. 2006;16(14):R516–R7. doi: 10.1016/j.cub.2006.06.044 16860721

[13] Li L, Yang X-J. Tubulin acetylation: responsible enzymes, biological functions and human diseases. Cellular and molecular life sciences. 2015;72:4237–55. doi: 10.1007/s00018-015-2000-5 26227334 PMC11113413

[14] Eshun-Wilson L, Zhang R, Portran D, Nachury MV, Toso DB, Löhr T, et al. Effects of α-tubulin acetylation on microtubule structure and stability. Proceedings of the National Academy of Sciences. 2019;116(21):10366–71. 10.1073/pnas.1900441116 PMC6535015 31072936

[15] Hubbert C, Guardiola A, Shao R, Kawaguchi Y, Ito A, Nixon A, et al. HDAC6 is a microtubule-associated deacetylase. Nature. 2002;417(6887):455–8. doi: 10.1038/417455a 12024216

[16] Li G, Jiang H, Chang M, Xie H, Hu L. HDAC6 α-tubulin deacetylase: a potential therapeutic target in neurodegenerative diseases. Journal of the neurological sciences. 2011;304(1–2):1–8. 21377170 10.1016/j.jns.2011.02.017

[17] Inoue T, Hiratsuka M, Osaki M, Oshimura M. The molecular biology of mammalian SIRT proteins: SIRT2 functions on cell cycle regulation. Cell Cycle. 2007;6(9):1011–8. 17457050 10.4161/cc.6.9.4219

[18] Forer A. Crane fly spermatocytes and spermatids: a system for studying cytoskeletal components. Methods in cell biology. 25: Elsevier; 1982. p. 227–52. doi: 10.1016/s0091-679x(08)61427-2 7109960

[19] Forer A. Characterization of the mitotic traction system, and evidence that birefringent spindle fibers neither produce nor transmit force for chromosome movement. Chromosoma. 1966;19(1):44–98. doi: 10.1007/BF00332793 5330243

[20] Amargant i Riera F, Barragan M, Vassena R, Vernos I. Insights of the tubulin code in gametes and embryos: from basic research to potential clinical applications in humans. Biol Reprod 2019; 100 (3): 575–589. 2019. 30247519 10.1093/biolre/ioy203

[21] de Pennart H, Houliston E, Maro B. Post-translational modifications of tubulin and the dynamics of microtubules in mouse oocytes and zygotes. Biology of the Cell. 1988;64(3):375–8. doi: 10.1016/0248-4900(88)90012-3 2906552

[22] Piperno G, LeDizet M, Chang X. Microtubules containing acetylated alpha-tubulin in mammalian cells in culture. The Journal of cell biology. 1987;104(2):289–302. doi: 10.1083/jcb.104.2.289 2879846 PMC2114420

[23] Schatten G, Simerly C, Asai DJ, Szöke E, Cooke P, Schatten H. Acetylated α-tubulin in microtubules during mouse fertilization and early development. Developmental biology. 1988;130(1):74–86. 3053299 10.1016/0012-1606(88)90415-0

[24] Wolf KW. Acetylation of α-tubulin in male meiotic spindles ofPyrrhocoris apterus, an insect with holokinetic chromosomes. Protoplasma. 1996;191:148–57.

[25] Forer A, Wilson PJ. Evidence that kinetochore fibre microtubules shorten predominantly at the pole in anaphase flea-beetle spermatocytes. Chromosome Research. 2000;8:151–63. doi: 10.1023/a:1009298620707 10780704

[26] Karamtzioti P, Ferrer-Vaquer A, Vernos I, Vassena R, Tiscornia G. Characterization of tubulin post translational modifications and their enzymes during human oocyte meiosis. Reproductive BioMedicine Online. 2024:103885. 10.1016/j.rbmo.2024.103885 39920028

[27] Wilson PJ, Forer A. Effects of nanomolar taxol on crane-fly spermatocyte spindles indicate that acetylation of kinetochore microtubules can be used as a marker of poleward tubulin flux. Cell Motil Cytoskeleton. 1997;37(1):20–32. doi: 10.1002/(SICI)1097-0169(1997)37:1&lt;20::AID-CM3&gt;3.0.CO;2-L 9142436

[28] LaFountain JR Jr, Cohan CS, Siegel AJ, LaFountain DJ. Direct visualization of microtubule flux during metaphase and anaphase in crane-fly spermatocytes. Molecular Biology of the Cell. 2004;15(12):5724–32. doi: 10.1091/mbc.e04-08-0750 15469981 PMC532050

[29] Schaap CJ, Forer A. Temperature effects on anaphase chromosome movement in the spermatocytes of two species of crane flies (Nephrotoma suturalis Loew and Nephrotoma ferruginea Fabricius). Journal of cell science. 1979;39(1):29–52. doi: 10.1242/jcs.39.1.29 528584

[30] Forer A, Pickett‐Heaps J. Fibrin clots keep non‐adhering living cells in place on glass for perfusion or fixation. Cell biology international. 2005;29(9):721–30. doi: 10.1016/j.cellbi.2005.04.010 16095930

[31] Butler KV, Kalin J, Brochier C, Vistoli G, Langley B, Kozikowski AP. Rational design and simple chemistry yield a superior, neuroprotective HDAC6 inhibitor, tubastatin A. Journal of the American Chemical Society. 2010;132(31):10842–6. doi: 10.1021/ja102758v 20614936 PMC2916045

[32] Vigushin DM, Ali S, Pace PE, Mirsaidi N, Ito K, Adcock I, et al. Trichostatin A is a histone deacetylase inhibitor with potent antitumor activity against breast cancer in vivo. Clin Cancer Res. 2001;7(4):971–6. 11309348

[33] Silverman‐Gavrila RV, Forer A. Effects of anti‐myosin drugs on anaphase chromosome movement and cytokinesis in crane‐fly primary spermatocytes. Cell motility and the cytoskeleton. 2001;50(4):180–97. doi: 10.1002/cm.10006 11807939

[34] Team RC. RA language and environment for statistical computing, R Foundation for Statistical. Computing. 2020.

[35] Lenth R, Bolker B, Buerkner P, Giné-Vázquez I, Herve M, Jung M, et al. emmeans: Estimated Marginal Means, aka Least-Squares Means (Version 1.8. 7)[Computer software]. 2023.

[36] Osborn M, Weber K. Immunofluorescence and immunocytochemical procedures with affinity purified antibodies: tubulin-containing structures. Methods in cell biology. 24: Elsevier; 1982. p. 97–132. 7048022 10.1016/s0091-679x(08)60650-0

[37] Wilson PJ, Forer A, Leggiadro C. Evidence that kinetochore microtubules in crane-fly spermatocytes disassemble during anaphase primarily at the poleward end. J Cell Sci. 1994;107 (Pt 11):3015–27. doi: 10.1242/jcs.107.11.3015 7699001

[38] Forer A, Otsuka S. Structural evidence for elastic tethers connecting separating chromosomes in crane-fly spermatocytes. Life Science Alliance. 2023;6(11). doi: 10.26508/lsa.202302303 37591724 PMC10435969

[39] Chen K, Xu L, Wiest O. Computational exploration of zinc binding groups for HDAC inhibition. The Journal of organic chemistry. 2013;78(10):5051–5. doi: 10.1021/jo400406g 23586590 PMC3703144

[40] Perdiz D, Mackeh R, Poüs C, Baillet A. The ins and outs of tubulin acetylation: more than just a post-translational modification? Cellular signalling. 2011;23(5):763–71. doi: 10.1016/j.cellsig.2010.10.014 20940043

[41] Haggarty SJ, Koeller KM, Wong JC, Grozinger CM, Schreiber SL. Domain-selective small-molecule inhibitor of histone deacetylase 6 (HDAC6)-mediated tubulin deacetylation. Proceedings of the National Academy of Sciences. 2003;100(8):4389–94. doi: 10.1073/pnas.0430973100 12677000 PMC153564

[42] Zilberman Y, Ballestrem C, Carramusa L, Mazitschek R, Khochbin S, Bershadsky A. Regulation of microtubule dynamics by inhibition of the tubulin deacetylase HDAC6. Journal of cell science. 2009;122(19):3531–41. doi: 10.1242/jcs.046813 19737819

[43] Sheykhani R, Berns M, Forer A. Elastic tethers between separating anaphase chromosomes in crane‐fly spermatocytes coordinate chromosome movements to the two poles. Cytoskeleton. 2017;74(2):91–103. doi: 10.1002/cm.21347 27935262

[44] Forer A, Berns MW. Elastic tethers between separating anaphase chromosomes regulate the poleward speeds of the attached chromosomes in crane-fly spermatocytes. Frontiers in Molecular Biosciences. 2020;7:161. doi: 10.3389/fmolb.2020.00161 32850955 PMC7405647

[45] North BJ, Marshall BL, Borra MT, Denu JM, Verdin E. The human Sir2 ortholog, SIRT2, is an NAD+-dependent tubulin deacetylase. Molecular cell. 2003;11(2):437–44. doi: 10.1016/s1097-2765(03)00038-8 12620231

[46] Vaziri H, Dessain SK, Ng Eaton E, Imai SI, Frye RA, Pandita TK, et al. hSIR2(SIRT1) functions as an NAD-dependent p53 deacetylase. Cell. 2001;107(2):149–59. doi: 10.1016/s0092-8674(01)00527-x 11672523

[47] Kite E, Forer A. The role of phosphorylation in the elasticity of the tethers that connect telomeres of separating anaphase chromosomes. Nucleus. 2020;11(1):19–31. doi: 10.1080/19491034.2019.1710329 31948316 PMC6973318

